# How much sugar is hidden in drinks marketed to children? A survey of fruit juices, juice drinks and smoothies

**DOI:** 10.1136/bmjopen-2015-010330

**Published:** 2016-03-21

**Authors:** Jane Boulton, Kawther M Hashem, Katharine H Jenner, Ffion Lloyd-Williams, Helen Bromley, Simon Capewell

**Affiliations:** 1 School of Dentistry, University of Liverpool, Liverpool, UK; 2 Wolfson Institute of Preventive Medicine, Queen Mary, University of London, London, UK; 3 Department of Public Health and Policy, University of Liverpool, Liverpool, UK

**Keywords:** PUBLIC HEALTH, SUGAR SWEETENED BEVERAGES, FOOD INDUSTRY, CHILDREN, QUALITATIVE RESEARCH

## Abstract

**Objective:**

To investigate the amount of sugars in fruit juices, juice drinks and smoothies (FJJDS) marketed to children.

**Design:**

We surveyed the sugars content (per 100 ml and standardised 200 ml portion) of all FJJDS sold by seven major UK supermarkets (supermarket own and branded products). Only products specifically marketed towards children were included. We excluded sports drinks, iced teas, sugar-sweetened carbonated drinks and cordials as being not specifically marketed towards children.

**Results:**

We identified 203 fruit juices (n=21), juice drinks (n=158) and smoothies (n=24) marketed to children. Sugars content ranged from 0 to 16 g/100 ml. The mean sugars content was 7.0 g/100 ml, but among the 100% fruit juice category, it was 10.7 g/100 ml. Smoothies (13.0 g/100 ml) contained the highest amounts of sugars and juice drinks (5.6 g/100 ml) contained the lowest amount. 117 of the 203 FJJDS surveyed would receive a Food Standards Agency ‘red’ colour-coded label for sugars per standardised 200 ml serving. Only 63 FJJDS would receive a ‘green’ colour-coded label. 85 products contained at least 19 g of sugars—a child's entire maximum daily amount of sugars. 57 products contained sugar (sucrose), 65 contained non-caloric sweeteners and five contained both. Seven products contained glucose-fructose syrup.

**Conclusions:**

The sugars content in FJJDS marketed to children in the UK is unacceptably high. Manufacturers must stop adding unnecessary sugars and calories to their FJJDS.

**Strengths and limitations of this study**

- A comprehensive assessment of the sugars content of fruit juice, juice drinks and smoothies (FJJDS) marketed to children.
- Assessment of standard portion size (200 ml) provided actual amount of sugars contained.
- Only FJJDS available at supermarket chains were recorded.
- New varieties of FJJDS appear in the market steadily and may be different.

## Introduction

Free sugars are the most important dietary cause of dental caries.^1^ ^2^ When consumed as a drink, fructose derived from fruit is as likely to cause dental caries as all other sugars.^3^ In the 2012 Children's Local Dental Health Survey, 28% of children aged 5 years in England had obvious decay in their primary teeth, rising to 35% of children aged 5 years in the North West.^4^ In 2013, 12% of children aged 3 years had experienced obvious dental decay.^5^ Dental decay is the most common reason for children in England being admitted to hospital.^6^ Furthermore, an increased intake of fructose and sugar-sweetened drinks has been associated with childhood obesity.^7^ ^8^

‘Free sugars’ refer to monosaccharides (such as glucose, fructose) and disaccharides (such as sucrose or table sugar) added to foods and drinks by the manufacturer, cook or consumer, and sugars naturally present in honey, syrups, fruit juices and fruit juice concentrates.^9^ It is a term used to distinguish between the sugars that are naturally present in fully unrefined carbohydrates such as fruit.

Consumption of free sugars, particularly in the form of beverages, can lead to an increase in total sugars intake and a reduction in the consumption of more nutritionally valuable food, thus leading to an unhealthy diet, increased weight and risk of non-communicable diseases.^10–12^ Most recent figures show one in five children aged 4–5 years and one in three children aged 10–11 years are overweight or obese.^6^ Children who are overweight are more likely to develop illnesses such as type 2 diabetes,^13^ have an increased likelihood of weight and health problems in adolescence and are more likely to become overweight or obese adults.^14^

Children aged between 4 and 10 years are now getting 30% of their sugars intake from soft drinks.^15^ In a recent survey of people's perception of sugars in drinks, the sugars content of fruit juices and smoothies was underestimated by 48% on average, whereas the sugars content of carbonated drinks was overestimated by 12%.^16^ Therefore, it is not surprising that the role of sugar-sweetened drinks and fruit juices in the increase in caries and obesity has recently been at the forefront of public debate.^17^ Furthermore, there is increasing public awareness of the negative impact of sugar-sweetened drinks on children's health.^18^ As a result, parents may replace sugar-sweetened carbonated drinks (that are perceived to be unhealthy) with fruit juices, juice drinks and smoothies (FJJDS) (that are perceived to be healthier). However, this can also have a negative health impact if the sugars content of FJJDS is equal to or higher than sugar-sweetened carbonated drinks.

This study aimed to record the sugars content of FJJDS marketed to children and determine which FJJDS contain sugar (sucrose), syrups, non-caloric sweeteners and free sugars derived from fruit.

Online supplementary appendix 1 provides definitions for the various types of sugars referred to in this paper.

10.1136/bmjopen-2015-010330.supp1 Supplementary appendix 1

## Methods

This survey looked at sugars per 100 ml and per standardised 200 ml portion of 203 soft FJJDS from seven supermarkets (Tesco, Asda, Sainsbury's, Marks & Spencer, Waitrose, The Co-operative and Morrisons) in the UK. FJJDS were identified by the definitions provided by the British Soft Drinks Association (box 1).

Box 1 **Fruit juice, juice drinks and smoothies as defined by the British Soft Drinks Association**

▸ Fruit juices are defined as, ‘100% pure juice made from the flesh of fresh fruit or from whole fruit, depending on the type used. It is not permitted to add sugars, sweeteners, preservatives, flavourings or colourings to fruit juice’

▸ Juice drinks are ‘1% to 99% juice, nectars, still flavoured waters, sports drinks and iced teas’.18a As neither sports drinks or iced teas are marketed towards children, they were not included in the survey

▸ Smoothies labelled as ‘fruit juice’ should not include any additional ingredients and are subject to the same regulations as fruit juice. Non-pure fruit smoothies may contain other ingredients such as yoghurt or milk, which must be labelled

Only products that were specifically marketed towards children were included. Sugar-sweetened drinks, sports drinks and iced teas were excluded. Cordials were also excluded. Although cordials are marketed towards children, they are not single-serve portions that were the focus of this survey. Cordials should be diluted one part cordial to nine parts water (with nutritional typical values shown with this dilution); however, people generally dilute to taste, so the sugar content of a single serve would vary from person to person. Furthermore, they are not suitable for lunchboxes.

Only single-portion cartons were recorded. This was determined using the supermarkets’ online shopping system and recording products under their children's FJJDS section—for example, ‘lunchbox and kids’. For supermarkets with no online shopping, size and design of packaging determined if the products were aimed towards children. Some drinks manufacturers produce products specifically designed for children—for example, Tropicana Kids, only products in these ranges were recorded. Both supermarket own and branded products were recorded.

Product sizes ranged from 65 to 500 ml and were recalculated at 200 ml from the 100 ml data. Teaspoons of sugar per standardised 200 ml portion (calculated using 4 g of sugar per teaspoon)^19^ were converted into teaspoons to help the researcher visualise the amount of sugar. The ingredient list was recorded for the analysis of sugar (sucrose), syrups, free sugars derived from fruit and non-caloric sweeteners (table 1).

**Table 1:** Identification of sugars and sweeteners translated into specific terms from the product ingredients list

Added sugar	Natural sugars	Non-caloric sweeteners
Sugar	Juice	Acesulfame K
Glucose-fructose syrup	Fruit juice from concentrate	Sucralose
		Sodium saccharin

Data were retrieved online or in-store between July and August 2014.

## Results

In total, 203 FJJDS were identified. Of the 203 products, 21 were fruit juices, 158 were juice drinks and 24 were smoothies (table 2).

**Table 2:** Number and average sugars content of products included

Category	Number	Mean sugars (g/100 ml)
Juice drinks	158	5.6
100% fruit juice	21	10.7
Smoothies	24	13.0
Total	203	7.0

Of the 179 fruit juices and juice drinks, 60 contained sugar (sucrose). Sugars content ranged from 0 to 16 g/100 ml. The average sugars content was 7.0 g/100 ml, but among the 100% fruit juice category, it was 10.7 g/100 ml. In total 117 of the 203 FJJDS surveyed would receive a Food Standards Agency (FSA) ‘red’ colour-coded label for sugars for a 200 ml serving, and only 63 FJJDS would merit a ‘green’ colour-coded label.^20^

Large variations existed in sugars between different types of FJJDS and within the same type of FJJDS. On average, juice drinks contained the lowest amounts (mean 5.6 g/100 ml) and smoothies contained the highest (mean 13.0 g/100 ml).

Of the 158 juice drinks, 85 contained at least 19 g of sugars—the child's entire maximum daily amount of free sugars.

A total of 60 products contained sugar (sucrose), 65 contained non-caloric sweeteners and 5 contained both. The majority of products were sweetened with acesulfame K, sucralose and aspartame. Of the 78/203 products, 12 contained sugars and non-caloric sweeteners. Of the 203 products, 7 contained glucose-fructose syrup. A total of 140 products contained no sugars or non-caloric sweeteners.

Online supplementary appendix 2 provides the full list of results.

10.1136/bmjopen-2015-010330.supp2 Supplementary appendix 2

## Discussion

The World Health Assembly (WHA) recently adopted a global target of 25% relative reduction in non-communicable diseases by 2025.^21^ This includes policies to reduce the content of sugars in non-alcoholic beverages, as well as to reduce the impact of marketing of non-alcoholic beverages high in sugars on children.^21^ In the UK, a national childhood obesity prevention strategy for England is expected in summer 2016. This is likely to include measures to reduce the consumption of sugar-sweetened carbonated soft drinks.^22^

Using a standardised 200 ml serving size, results from this survey show 64% of FJJDS surveyed would contain at least half of a young child's maximum sugar intake for the day (9.5 g), with 42% of products containing a full days recommended sugars allowance (19 g for children aged 4–6 years).^23^ Current guidelines state a 150 ml glass of pure fruit juice at meal times counts as a maximum of one of the ‘5 a Day’.^24^ However, Public Health England is aiming to refresh the ‘5 a Day’ campaign, including a reconsideration of the advice on fruit juice and smoothies.^25^ This appears crucial, based on our findings. Of the products included in this survey, only five produced products at a 150 ml size. All other products exceeded the recommended serving size and are likely to be consumed in a single seating as a single portion, therefore greatly increasing the child's sugars intake.

Furthermore, the FSA thresholds relate to adult intake of sugars; therefore, we can speculate that the number of FJJDS with a ‘red’ colour code is an underestimation and the number of FJJDS with a ‘green’ colour code is an overestimation for children's sugars consumption.^20^

WHO recently revised guidelines recommending reducing total daily energy intake from free sugars from 10% to below 5%.^19^ This has now been supported by the government's Scientific Advisory Committee on Nutrition (SACN).^23^ SACN recommends that the average population intake of free sugars should not exceed 5% of total dietary energy for age groups from 2 years upwards.^23^ This has been endorsed by the UK government. For young children, this 5% limit will represent a maximum of around 19 g/day (nearly five teaspoons of sugar).^19^ A recent critical in-depth review goes further, recommending that an intake below 2–3% is necessary to avoid dental caries. The review suggests that an increase from near-zero free sugars to 5% of energy intake doubles the prevalence of caries in children.^26^ Figures from the latest UK National Diet and Nutrition Survey data show that in children aged 4–10 years, the average intake of free sugars is 14.7% of total energy^27^—threefold higher than the new recommendations.

One key difference between whole fruit and juice is fibre content. Whole fruit slows down consumption and has a satiating effect.^28^ Research shows the body metabolises fruit juice in a different way compared to whole fruit. After whole fruit consumption, the body seems to adjust its subsequent energy intake appropriately, whereas after fruit juice consumption, the body does not compensate for the energy intake.^29^

We did not initially set out to record the number of products containing non-caloric sweeteners. However, of the 203 FJJDS included in our study, 78 contained non-caloric sweeteners, the majority of which were sweetened with acesulfame K, sucralose and aspartame. Although all these non-caloric sweeteners have been declared safe by the European Food Safety Authority,^30^ health experts believe a reduction in the overall sweetness of products is necessary in order for children's taste to become less accustomed to sweetness.^31^ Although replacing sugars with non-caloric sweeteners may lead to improved markers of long-term metabolic health and weight loss, it will not necessarily provide a long-term solution to reducing sugar consumption and subsequent health problems.

All labels on products recorded contained a reference intake, in line with European law.^32^ The reference intake provides one figure, which applies to an average-sized adult female doing an average amount of physical activity.^33^ This figure is not appropriate for children who have very different energy intake requirements and energy expenditure. Furthermore, pure fruit juices are labelled as Fruit Juice, while products with any other added ingredients are known as Juice Drinks. With all this in mind, it is fair to say that labelling of fruit juice and juice drinks marketed towards children can be unclear and thus potentially confusing for parents trying to make healthier choices.

### Study limitations

We acknowledge a number of limitations to this study. First, only FJJDS available at supermarket chains were recorded, but there may be other products available to the public in other smaller independent stores. Second, new varieties of FJJDS appear in the market steadily and may have different properties from those recorded. Results, comparisons or conclusions drawn from this survey can thus only apply to the products in this time period (July and August 2014). Third, we only focused on sugars content and did not include an assessment of the portion size of beverages marketed to children, which could be considerably larger than the recommended amount. Fourth, no lactose added to food or drink is now considered as free sugars. However, in the products where some milk was added, we could not identify from the labelling what percentage came from the lactose, therefore it was not excluded. Finally, the study was based on sugars content data provided on the available packaging labels in store on the dates of collection; hence, we relied on the accuracy of the data provided on the label. Therefore, it is assumed that the manufacturers provide accurate and up-to-date information in line with EU Regulations. However, future studies should include free sugars determined through laboratory analysis to achieve a better understanding of the true free sugars content. Nevertheless, the results of this study are relevant and serve to document the sugar content of FJJDS products sold in the UK supermarkets, providing a foundation for future studies and evidence for the sugar reduction programme and the soft drink industry.

## Conclusions

The sugars content in FJJDS marketed to children in the UK is high. Over 40% of products surveyed contained at least 19 g of sugars—a child's entire maximum daily amount of free sugars.

We suggest that FJJDS with high free sugars content should not count as one of the UK government's ‘5 a Day’ recommendations. Ideally, fruit should be consumed in its whole form, not as juice. Parents should dilute fruit juice with water, opt for unsweetened juices and only give them during meals. Portions should be limited to 150 ml a day. In order to help combat the growing problem of childhood obesity, manufacturers need to stop adding unnecessary sugars and calories to their FJJDS now; otherwise, it will be essential for the government to introduce legislation to regulate the free sugars content of these products.
