# Supplementary material for: How much sugar is hidden in drinks marketed to children? A survey of fruit juices, juice drinks and smoothies
Source: BMJ Open. 2016 Mar 21;6(3):e010330. doi: 10.1136/bmjopen-2015-010330 (PMC4809069; doi:10.1136/bmjopen-2015-010330)
Supplement: Supplementary appendix 1 [file bmjopen-2015-010330supp_appendix1.pdf]

APPENDIX 1 DEFINITIONS

Free sugars (sugars) comprise all monosaccharides* and disaccharides* added to foods by the manufacturer, cook or consumer, plus sugars naturally present in honey, syrups and unsweetened fruit juices. Under this definition lactose (the sugar in milk) when naturally present in milk and milk products and the sugars contained within the cellular structure of foods (particularly fruits and vegetables) are excluded.
*Monosaccharides are single sugar units (glucose and fructose) and disaccharides are two single units joined together (sucrose)

Fructose or fruit sugar is a simple ketonic monosaccharide found in many plants, where it is often bonded to glucose to form the disaccharide sucrose.

Glucose fructose syrup is a liquid sweetener used in the manufacturing of foods and beverages.

Non-caloric sweeteners are substances that are used in place of caloric sweeteners such as sugar. They may also be called sugar substitutes, nonnutritive sweeteners (NNS), and artificial sweeteners. They include Aspartame, Sucralose, Saccharin, Stevia, Acesulfame K, Neotame and Cyclamates.

Sucrose (sugar) is a crystalline sugar found in many plants, especially sugar cane and sugar beet.
