# Supplementary material for: How much sugar is hidden in drinks marketed to children? A survey of fruit juices, juice drinks and smoothies
Source: BMJ Open. 2016 Mar 21;6(3):e010330. doi: 10.1136/bmjopen-2015-010330 (PMC4809069; doi:10.1136/bmjopen-2015-010330)
Supplement: Supplementary appendix 2 [file bmjopen-2015-010330supp_appendix2.pdf]

APPENDIX 2 – FULL RESULTS

Data table sorted by category, alphabetically, highest sugars (g) per 100ml. Product information was collected online, instore or direct from manufacturers.

Colour coding based on new front of pack colour-coded nutrition labelling criteria.

Sugars - Red >13.5g/portion or >11.25g/100ml, Amber >2.5≤11.25/100ml, Green ≤2.25g/100ml

Serving size has been standardised to 200ml, the standard portion size given on 46% of packs. Although many varieties are available in a 200ml carton size, serving size ranged from 85-500ml and have been recalculated as 200ml for comparison purposes.

Product Name	Portion size	Sugars (g) per 100ml	Sugars (g) per 200ml serving*	Teaspoons sugar (g) equiv/ 200ml serving*
**Fruit Juice**				
Asda Chosen by Kids Tropical Juice From Concentrate 4 x180ml	180	13.0	26.0	7
Morrisons Pineapple Juice From Concentrate 3 x 200ml	200	12.6	25.2	6
Eager Cloudy Pressed Apple & Mango Juice 4 x 180ml	180	12.0	24.0	6
Essential Waitrose Pure Pineapple Juice From Concentrate 4 x 200ml	200	11.9	23.8	6
Eager 100% Cloudy Pressed Pineapple Juice 4 x 180ml	180	11.5	23.0	6
Morrisons Apple Juice From Concentrate 3 x 200ml	200	11.4	22.8	6
Calypso 12 Juice Shots 100% Pure Apple Juice From Concentrate 12 x 85ml	85	11.4	22.8	6
Essential Waitrose Pure Apple Juice From concentrate 3 x 200ml	200	11.2	22.4	6
Jucee 100% Pure Pineapple Juice From Concentrate 6 x 200ml	200	11.0	22.0	6
The Co-operative Loved By Us Pure Pineapple Juice From Concentrate 3 x 200ml	200	11.0	22.0	6
Jucee 100% Pure Apple Juice From Concentrate 6 x 200ml	200	10.4	20.8	5

Jucee 100% Pure Cherries & Berries 6 x 200ml	200	10.4	20.8	5
Morrisons Orange Juice from Concentrate 3 x 200ml	200	10.4	20.8	5
Sainsbury's Smooth Orange Juice from Concentrate 3 x 200ml	200	10.4	20.8	5
The Co-operative Pure Apple Juice from Concentrate 3 x 200ml	200	10.4	20.8	5
Copella English Apple 330ml	330	10.3	20.6	5
Essential Waitrose Pure Orange Juice From Concentrate 6 x 200ml	200	10.2	20.4	5
Jucee 100% Pure Orange Juice From Concentrate 6 x 200ml	200	9.2	18.4	5
The Co-operative Loved By Us Pure Orange Juice From Concentrate 3 x 200ml	200	9.2	18.4	5
Innocent 100% Tropical Juice for Kids 4x180ml	180	8.9	17.8	4
Innocent 100% Apple Juice for Kids 4 x 180ml	180	7.8	15.6	4
		average	21.4	5
**Juice Drinks**				
Rubicon Lychee Exotic Juice Drink 288ml	288	13.3	26.6	7
Rubicon Passion Exotic Juice Drink 288ml	288	13.1	26.2	7
Aasani Passion Fruit 250ml	250	12.8	25.6	6
SunStream Splash Mango Juice Drink 10 x 200ml	200	12.7	25.4	6
Rubicon Mango Exotic Juice Drink 288ml	500	12.6	25.2	6
Rubicon Guava Exotic Juice Drink 288ml	288	12.6	25.2	6
Aasani Tropical Juice Drink 250ml	250	12.5	25.0	6
Tesco Blackcurrant Pouch Juice Drink 10X200ml	200	12.5	25.0	6
Capri-Sun Blackcurrant Juice Drink	200	12.2	24.0	6
Sainsbury's Fruit Slurps Blackcurrant & Apple Juice Drink pouches 10 x 200ml	200	11.7	23.4	6
Ocean Spray Cranberry &R/Berry Juice Drink 3X200ml	200	11.6	23.2	6
SunStream Splash Lychee Juice Drink 10 x 200ml	200	11.6	23.2	6
Rubicon Pomegranate Exotic Juice Drink 288ml	288	11.3	22.6	6
Sainsbury's Apple Juice from Concentrate 3 x 200ml	200	11.3	22.6	6
Rubicon Papaya Exotic Juice Drink 288ml	288	11.1	22.2	6
Capri-Sun Orange & Tropical 330ml	330	11.0	22.0	6
Robinsons Fruit Shoot Apple & Blackcurrant 275ml	275	11.0	22.0	6

Ocean Spray Cranberry Classic 3 X 200Ml	200	11.0	22.0	6
SunStream Splash Tropical Juice Drink 200ml	200	11.0	22.0	6
Tesco Fruit Splash Apple Juice Drink 10X200ml	200	11.0	22.0	6
Ribena Strawberry 10 x 200ml	200	10.9	21.8	5
Sainsbury's Fruit Slurps Orange Juice Drink pouches 10 x 200ml	200	10.9	21.8	5
Um Bongo Mango and Apple Congocoction Juice Drink 200ml	200	10.9	21.8	5
Sunny D Zingy Orange Berry Juice Drink 500ml	500	10.6	21.2	5
Capri-Sun Juice Drink Tropical 10 x 200ml Pouches	200	10.6	21.2	5
Waitrose Apple & Mango Juice Drink 5 x 250ml	250	10.5	21.0	5
Ribena Blackcurrant 10 x 200ml	200	10.5	21.0	5
Ribena Raspberry 288ml	288	10.4	20.8	5
Capri-Sun Apple Juice Drink 10 x 200ml	200	10.4	20.8	5
Waitrose Orange & Passionfruit Fruit Juice Drink 5 x 250ml	250	10.3	20.6	5
Sunny D Juicy Orange Passion Juice Drink 5 x 200ml	200	10.3	20.6	5
Capri - Sun Juice Drink Summer Berries 200ml	200	10.2	20.4	5
SunStream Splash Orange Juice Drink 10 x 200ml	200	10.2	20.4	5
SunStream Splash Apple Juice Drink 10 x 200ml	200	10.1	20.2	5
Capri-Sun Apple & Blackcurrant Juice Drink 330ml	330	10.0	20.0	5
Capri-Sun Orange 3 x 330ml	330	10.0	20.0	5
Robinsons Fruit Shoot Orange 275ml	275	10.0	20.0	5
Capri-Sun Juice Drink Orange 10 x 200ml Pouches	200	10.0	20.0	5
Um Bongo Orang-Utan Orange Juice Drink 200ml	200	10.0	20.0	5
Ribena Pineapple & Passion Fruit 500ml	500	9.9	19.8	5
Um Bongo Original Congocoction Juice Drink 3 x 200ml	200	9.9	19.8	5
Ribena Apple 500ml	500	9.8	19.6	5
Ribena Mango & Lime 500ml	500	9.8	19.6	5
Don Simon Disney-Pixar Monsters University Tropical Juice Drink 200ml	200	9.8	19.6	5
Capri - Sun Juice Drink Mango	200	9.6	19.2	5
Capri-Sun 100% Pure Juice Apple 5 x 200ml Pouches	200	9.3	18.6	5

Robinsons Fruit Shoot My-5 Apple & Pear 200ml	200	9.0	18.0	5
The Little Crackers Drinks Co Apple and Raspberry Juice Drink 3 x 200ml	200	8.9	17.8	4
Sainsbury's Fruit Slurps Tropical Juicy Water 3 x 250ml	250	8.7	17.4	4
Cherrygood Burst 3X200ml	200	8.6	17.2	4
Don Simon Disney-Pixar Monsters University Apple & Blackcurrant Juice Drink	200	8.6	17.2	4
Robinsons Fruit Shoot My-5 Orange & Pineapple 200ml	200	8.6	17.2	4
Sunny D Smooth California Citrus Juice Drink 500ml	500	8.5	17.0	4
Capri-Sun Apple & Cherry Juice Drink 10 x 200ml	200	8.4	16.8	4
Feel Good Kids Juice Drink Orange, Pineapple and Banana 4 x 180ml	180	8.4	16.8	4
Ribena Orange 288ml	288	8.2	16.4	4
Robinsons Fruit Shoot My-5 Apple & Blackcurrant 200ml	200	8.2	16.4	4
Sunny Delight Florida Style Orange Drink 5 x 200ml	200	8.2	16.4	4
Capri-Sun Fruit Crush Apple & Blackcurrant Juice Drink 5 x 200ml	200	8.1	16.2	4
The Little Crackers Drinks Co Orange and Lemon Juice Drink 3 x 200ml	200	8.1	16.2	4
Capri-Sun Fruit Crush Tropical 75% Fruit Juice 25% Spring Water	200	8.0	16.0	4
5 Alive Citrus Five Fruit Blend 150ml	150	8.0	16.0	4
Sainsbury's Fruit Slurps Apple & Mango Juicy Water 3 x 250ml	250	7.9	15.8	4
Capri-Sun Fruit Crush Apple & Pear Juice Drink 200ml	200	7.9	15.8	4
The Little Crackers Drinks Co Blackcurrant and Cherry Juice Drink 3 x 200ml	200	7.9	15.8	4
Sainsbury's Fruit Slurps Apple, Raspberry & Grape Juicy Water 3 x 250ml	250	7.8	15.6	4
Tropicana Kids Apple Juice Drink 4 x 200ml	200	7.8	15.6	4
Tropicana Kids Orange Juice Drink 4 x 200ml	200	7.7	15.4	4
The Little Crackers Drinks Co Pear and Vanilla Juice Drink 3 x 200ml	200	7.2	14.4	4
Feel Good Kids Juice Drink Blackcurrant, Apple & Grape 4 x 180ml	180	7.2	14.4	4
I Mune Nurture Orange & Pineapple Water 4X200ml	200	7.0	14.0	4
Cawston Press Kids' Blend Apple & Mango 3 x 200ml	200	6.8	13.6	3
Vimto 500ml	500	6.5	13.0	3
Cawston Press Kids' Blend Apple & Pear 3 x 200ml	200	6.4	12.8	3
Heinz Apple and Blackcurrent Fruity Spring Water	150	6.4	12.8	3

5 Alive Berry Five Fruit Blend Juice Drink 250ml	250	6.1	12.2	3
5 Alive Tropical Five Fruit Blend Juice Drink 250ml	250	6.1	12.2	3
5 Alive Apple 150ml	150	6.1	12.2	3
Peter Rabbit Organics Organic Pear Juice Drink 150ml	150	6.1	12.2	3
Drench Juicy Spring Water Orange and Passion Fruit 440ml	440	6.0	12.0	3
Innocent Apples and Peaches Fruity Water for Kids 180ml	180	6.0	12.0	3
Calypso Aqua Juice Apple Juicy Mineral Water 200ml	200	5.7	11.4	3
Calypso Aqua Juice Forest Fruits Juicy Mineral Water 200ml	200	5.6	11.2	3
Calypso Aqua Juice Orange Juicy Mineral Water 200ml	200	5.4	10.8	3
Innocent Apples and Mangoes Fruity Water for Kids 180ml	180	5.0	10.0	3
Peter Rabbit Organics Organic Juice with Water Summer Berries 250ml	250	4.7	9.4	2
Peter Rabbit Organics Organic Juice with Water Orange and Mango 250ml	250	4.5	9.0	2
Peter Rabbit Organics Organic Apple and Grape Juice Drink 150ml	150	4.4	8.8	2
Peter Rabbit Organics Organic Blackcurrant and Apple Juice Drink 150ml	150	4.2	8.4	2
Oasis Blackcurrant Apple 500ml	500	4.1	8.2	2
Oasis Mango Medley 500ml	500	4.1	8.2	2
Oasis Pineapple Rush 500ml	500	4.1	8.2	2
Oasis Citrus Punch 375ml	375	4.1	8.2	2
Oasis Summer Fruits 6 x 375ml	375	4.1	8.2	2
Rubicon Mango Light 288ml	288	3.8	7.6	2
Del Monte Fruit Burst Sweet Apple & Blackcurrant 200ml	200	1.9	3.8	1
Moshi Water Strawberry & Raspberry 4X250ml	250	1.9	3.8	1
Moshi Water Blackcurrant 4 X 250Ml	250	1.7	3.4	1
Del Monte Fruit Burst Fruity Pineapple & Raspberry 6 x 200ml	200	1.7	3.4	1
Del Monte Fruit Burst Cool Cherry & Luscious Lime 200ml	200	1.6	3.2	1
Del Monte Fruit Burst Sour Apple & Strawberry 200ml	200	1.6	3.2	1
Del Monte Fruit Burst Sherbet Lemon Orange & Lime 6 x 200ml	200	1.3	2.6	1
Morrisons Nas Apple & Blackcurrant Juice 3 x 250ml	250	1.2	2.4	1
Asda Chosen by Kids Apple & Pear Juice Drink 5 x 200ml	200	1.2	2.4	1

Tesco Fruit Splash Orange 4X250ml	250	1.1	2.2	1
Asda Chosen by Kids Apple & Mango Juice Drink 5 x 200ml	200	1.1	2.2	1
Asda Chosen by Kids Cherries & Berries Juice Drink 5 x 200ml	200	1.1	2.2	1
Asda Chosen by Kids Pineapple & Orange Juice Drink 5 x 200ml	200	1.1	2.2	1
Asda Chosen by Kids No Added Sugar Apple & Blackcurrant Juice Drinks 6 x 300ml	300	1.0	2.0	1
Robinsons Fruit Shoot Juice Drink Orange 300ml	300	1.0	2.0	1
Jucee Apple and Blackcurrant Juice Drink 4 x 250ml	250	1.0	2.0	1
Jucee Cherries & Berries Juice Drink 4 x 250ml	250	1.0	2.0	1
Morrisons No Added Sugar Apple Juice Drink 3 x 250ml	250	1.0	2.0	1
Sainsbury's Fruit Slurps Apple Juice Drink 8 x 250ml	250	1.0	2.0	1
Sainsbury's Fruit Slurps Blackcurrant & Apple Juice Drink 4 x 250ml	250	1.0	2.0	1
Tesco Apple & Blackcurrant Juice Drink No Added Sugar 3X250ml	250	1.0	2.0	1
Tesco Apple Juice Drink No Added Sugar 3X250ml	250	1.0	2.0	1
Tesco Tropical Juice No Added Sugar 3X250ml	250	1.0	2.0	1
Asda Chosen by Kids Apple & Blackcurrant Juice Drink 5 x 200ml	200	1.0	2.0	1
Ribena Plus Immunity Support No Added Sugar Apple & Peach 10 x 200ml	200	1.0	2.0	1
Asda Chosen by Kids No Added Sugar Orange Juice Drinks 6 x 300ml	300	0.9	1.8	0
Asda Chosen by Kids No Added Sugar Tropical Juice Drinks 6 x 300ml	300	0.9	1.8	0
Jucee No Added Sugar Orange Juice Drink 250ml	250	0.9	1.8	0
Sainsbury's No Added Sugar Fruit Slurps Orange Juice Drink 4 x 250ml	250	0.9	1.8	0
Sainsbury's No Added Sugar Fruit Slurps Summer Fruits Juice Drink 8 x 250ml	250	0.9	1.8	0
Ribena Plus Immunity Support No Added Sugar Red Apple 4 x 200ml	200	0.9	1.8	0
Ribena Plus Immunity Support No Added Sugar Summer Fruits	200	0.9	1.8	0
Robinsons Fruit Shoot Apple 8 x 200ml	200	0.9	1.8	0
Asda Chosen by Kids No Added Sugar Summer Fruits Juice Drinks 6 x 300ml	300	0.8	1.6	0
Robinsons Fruit Shoot Tropical 275ml	275	0.8	1.6	0
Robinsons Fruit Shoot Apple & Blackcurrant 15 x 200ml	200	0.8	1.6	0
Robinsons Fruit Shoot Orange 15 x 200ml	200	0.8	1.6	0
Vimto Cherry No Added Sugar 3 x 250ml	250	0.7	1.4	0

Robinsons Fruit Shoot Summer Fruits 8 x 200ml	200	0.7	1.4	0
Oasis Peach Passionfruit Light 500ml	500	0.6	1.2	0
Oasis Summer Fruits Light 500ml	500	0.5	1.0	0
Ribena No Added Sugar Blackcurrant 288ml	288	0.5	1.0	0
Ribena Plus Healthy Bones No Added Sugar Raspberry & Apple 10 x 200ml	200	0.5	1.0	0
Vimto No Added Sugar 500ml	500	0.4	0.8	0
Ribena Plus Healthy Bones No Added Sugar Blackcurrant 10 x 200ml	200	0.4	0.8	0
Sainsbury's Basics No Added Sugar Apple Juice Drink 9 x 250ml	250	0.3	0.6	0
Sainsbury's Basics No Added Sugar Orange Juice Drink 9 x 250ml	250	0.3	0.6	0
Vimto Strawberry No Added Sugar 3 x 250ml	250	0.3	0.6	0
Calypso Cups Disney-Pixar Monsters University Juice Drinks 24 x 185ml	185	0.3	0.6	0
Ribena Plus Apple & Peach 500ml	500	0.2	0.4	0
Panda Blackcurrant Still Juice Drink 330ml	330	0.2	0.4	0
Panda Orange Still Juice Drink 330ml	330	0.2	0.4	0
Panda Raspberry Still Juice Drink 6 x 330ml	330	0.2	0.4	0
Calypso Disney-Pixar Monsters University Apple Juice Drink 3 x 200ml	200	0.2	0.4	0
Calypso Disney Orange Juice Drink 6 x 200ml	200	0.1	0.2	0
Calypso Disney Princess Blackcurrant Juice Drink 6 x 200ml	200	0.1	0.2	0
Calypso Disney-Pixar Orange Juice Drink 3 x 200ml	200	0.1	0.2	0
Robinsons Fruit Shoot Mega Hydro Spring Water Drink Apple & Raspberry 4 x 350ml	250	0.0	0.0	0
Robinsons Fruit Shoot Hydro Mini Spring Water Drink Blackcurrant 8 x 200ml	200	0.0	0.0	0
Robinsons Fruit Shoot Hydro Mini Spring Water Drink Orange & Pineapple 8 x 200ml	200	0.0	0.0	0
Asda Chosen by Kids No Added Sugar Cool Cups Orange / Raspberry 24 x 185ml	185	0.0	0.0	0
Asda Chosen by Kids No Added Sugar Cool Cups Strawberry, Apple & Blackcurrant 24 x 185ml	185	0.0	0.0	0
Sainsbury's Eric The Elephant Fruit Cup Drink 12x185ml	185	0.0	0.0	0
		average	11.1	

**Smoothies**				
Tesco Goodness Slurper Apple & Banana Fruit Smoothie Snack for kids	90	16.1	32.2	8
Tesco Goodness Slurper Apple & Strawberry Fruit Smoothie Snack for kids	90	16.1	32.2	8
Tesco Goodness Slurper Apple Fruit Smoothie Snacks	90	16.1	32.2	8
Ella's Kitchen The Yellow One Squished Smoothie Fruits 5 x 90g	90	15.1	30.2	8
Innocent Smoothies for Kids – Apples and Blackcurrants, 6 x 180ml	180	14.2	28.4	7
Ella's Kitchen The Orange One Squished Smoothie Fruits 5 x 90g	90	14.2	28.4	7
Ella's Kitchen The Purple One Squished Smoothie Fruits 5 x 90g	90	13.8	27.6	7
Sainsbury's Smoothie, Apple & Blackcurrant 5x90g	90	13.8	27.6	7
Don Simon Disney Pixar Cars 2 Tropical Smoothie 3 x 200ml	200	13.7	27.4	7
Don Simón Don Simon My Little Smoothie Tropical 3 x 200ml	200	13.7	27.4	7
Ella's Kitchen The Green One Squished Smoothie Fruits 5 x 90g	180	13.1	26.2	7
Innocent Smoothies for Kids Oranges, Mangoes & Pineapples 6 x 180ml	180	13.1	26.2	7
Don Simón Don Simon My Little Smoothie Peach & Passionfruit 3 x 200ml	200	13.0	26.0	7
Happy Monkey Smoothie Strawberry & Banana 4 x 180ml	180	12.7	25.4	6
Don Simon Disney Princess Strawberry & Raspberry Smoothie 3 x 200ml	200	12.6	25.2	6
Don Simón Don Simon My Little Smoothie Strawberry & Raspberry 3 x 200ml	200	12.6	25.2	6
Happy Monkey Smoothie Orange & Mango 4 x 180ml	180	12.1	24.2	6
Innocent Smoothies for Kids Cherries and Strawberries 4 x 180ml	180	11.5	23.0	6
Ella's Kitchen The Red One Squished Smoothie Fruits 5 x 90g	90	11.5	23.0	6
Tesco Goodness Slurpers Apple & Blackcurrant Fruit Smoothie Snacks	90	11.5	23.0	6
Tesco Goodness Slurpers Tropical Fruit Smoothie Snacks	90	11.5	23.0	6
Lake Distict Frumoo Raspberry & Blueberry 230ml	200	10.7	21.4	5
Innocent 100% Pure Fruit Smoothie For Kids Strawberries, Blackberries & Raspberries 180ml	180	9.5	19.0	5
The Co-operative Strawberry Smoothie	250	9.4	18.8	5
		average	26.0	6
